# Developmental prediction modeling based on diffusion tensor imaging uncovering age-dependent heterogeneity in early childhood autistic brain

**DOI:** 10.1186/s13229-023-00573-2

**Published:** 2023-10-30

**Authors:** Xinyue Huang, Yating Ming, Weixing Zhao, Rui Feng, Yuanyue Zhou, Lijie Wu, Jia Wang, Jinming Xiao, Lei Li, Xiaolong Shan, Jing Cao, Xiaodong Kang, Huafu Chen, Xujun Duan

**Affiliations:** 1 The Clinical Hospital of Chengdu Brain Science Institute, School of Life Science and Technology, University of Electronic Science and Technology of China, Chengdu, 610054 People’s Republic of China; 2 MOE Key Lab for Neuro Information, High-Field Magnetic Resonance Brain Imaging Key Laboratory of Sichuan Province, University of Electronic Science and Technology of China, Chengdu, 610054 People’s Republic of China; 3 Department of Medical Psychology, The First Affiliated Hospital, Hainan Medical University, Haikou, 571199 Hainan People’s Republic of China; 4 Department of Children’s and Adolescent Health, Public Health College of Harbin Medical University, Harbin, 150086 People’s Republic of China; 5 Child Rehabilitation Unit, Affiliated Sichuan Provincial Rehabilitation Hospital of Chengdu University of TCM, Sichuan Bayi Rehabilitation Center, Chengdu, 611135 People’s Republic of China

## Abstract

**Objective:**

There has been increasing evidence for atypical white matter (WM) microstructure in autistic people, but findings have been divergent. The development of autistic people in early childhood is clouded by the concurrently rapid brain growth, which might lead to the inconsistent findings of atypical WM microstructure in autism. Here, we aimed to reveal the developmental nature of autistic children and delineate atypical WM microstructure throughout early childhood while taking developmental considerations into account.

**Method:**

In this study, diffusion tensor imaging was acquired from two independent cohorts, containing 91 autistic children and 100 typically developing children (TDC), aged 4–7 years. Developmental prediction modeling using support vector regression based on TDC participants was conducted to estimate the WM atypical development index of autistic children. Then, subgroups of autistic children were identified by using the k-means clustering method and were compared to each other on the basis of demographic information, WM atypical development index, and autistic trait by using two-sample t-test. Relationship of the WM atypical development index with age was estimated by using partial correlation. Furthermore, we performed threshold-free cluster enhancement-based two-sample t-test for the group comparison in WM microstructures of each subgroup of autistic children with the rematched subsets of TDC.

**Results:**

We clustered autistic children into two subgroups according to WM atypical development index. The two subgroups exhibited distinct developmental stages and age-dependent diversity. WM atypical development index was found negatively associated with age. Moreover, an inverse pattern of atypical WM microstructures and different clinical manifestations in the two stages, with subgroup 1 showing overgrowth with low level of autistic traits and subgroup 2 exhibiting delayed maturation with high level of autistic traits, were revealed.

**Conclusion:**

This study illustrated age-dependent heterogeneity in early childhood autistic children and delineated developmental stage-specific difference that ranged from an overgrowth pattern to a delayed pattern.

*Trial registration* This study has been registered at ClinicalTrials.gov (Identifier: NCT02807766) on June 21, 2016 (https://clinicaltrials.gov/ct2/show/NCT02807766).

**Supplementary Information:**

The online version contains supplementary material available at 10.1186/s13229-023-00573-2.

## Introduction

Autism is an early-onset neurodevelopmental condition that is diagnosed on the basis of the following essential features: persistent challenges in social communication and social interaction and the presence of restricted, repetitive behaviors, interests, or activities. Autistic children exhibit the most evident characteristic usually in early childhood and early school years, with some characteristics in some areas being promoted in later childhood due to learning and compensation [1]. The human brain undergoes rapid growth during pre-school years and reaches approximately 90% of its adult volume by the age of 6 years [2]. Thus, this period of time appears to be crucial for the precise understanding of the pathogenesis of autistic people before latter life events confound the interpretation of autistic characteristics.

In the developing brain, axonal development and myelination are key processes promoting the speed and energy efficiency of nerve conduction [3]. Deviation in these processes may cause challenges in information processing [4, 5], which have been frequently reported in autistic people [6–9]. Atypical axon and myelin properties have been reported in mouse models of autism and postmortem autistic brain [10–16]. In parallel, in vivo evidence using diffusion tensor imaging (DTI) suggested atypical microstructure in a number of white matter (WM) tracts and its association with core characteristic in autistic people [17, 18]. The individual variation in WM microstructure was inferred to be associated with changes in myelination and axon number and size [19]. Thus, the elaboration of axon and myelin growth could be essential for understanding the neurodevelopment of autistic people. Previous studies reported that preadolescent autistic children exhibited atypical overgrowth of brain structure and function, whereas older autistic people showed an inverse developmental pattern [20–23]. However, the age-dependent heterogeneity of atypical WM microstructure during the early progression of autistic people is unclear.

Diffusion properties have been widely used to track human brain development in vivo. A longitudinal study of typically developing children (TDC) aged 4–11 years reported significant linear increase in global fractional anisotropy (FA) as well as decrease in mean diffusivity (MD) and radial diffusivity (RD) over time, suggesting a linear developmental pattern of WM microstructures [24]. In early childhood, myelin rapidly develops, with cognitive abilities dramatically increasing at the same time [25]. This is concordant with the results of studies on WM development using DTI, which delineated a rapid change in diffusion properties during early development [26, 27] and reported a tract-specific association between expressive language and cognitive abilities [28]. In this regard, the age-dependent nature of brain development should be carefully considered while attempting to reveal atypical WM microstructures of autistic children underlying the developing brain.

One of the most commonly used methods for investigating atypical WM microstructures in autistic people is the case–control method [29–31], which could enable efficient and direct observation between groups. However, it might introduce potential confounding bias and thus may not be suitable for investigating differences that are inconstant during the time course of brain development [32]. Another commonly used method is the linear mixed effects model [6, 23, 33, 34], which can explore group interaction across development. However, the dependent variable of this model requires information about the certain varied brain regions of the condition. Such a requirement might result in an incomplete understanding of the condition because it heavily depends on the prior knowledge. In this regard, exploring the extent of atypical neurodevelopment in autistic children warrants the elaboration of typical brain development, such as brain age models which can generate an interpretable score from complex multivariate pattern, and normative models which can estimate the individual difference with respect to an expected pattern [35–41]. Focusing on the mismatches between the brain-derived predicted age and true chronological age, brain age models proposed approach using multivariate regression to predict age from a pattern of brain-derived measures [38–41]. In the developing population, estimating the individual deviation from a time-varying perspective may potentially demonstrate the age-dependent heterogeneity that exists in development.

In this study, we aimed to quantify the atypical development of WM microstructures in autistic children on the basis of developmental prediction modeling. We hypothesized that the autistic children might exhibit heterogeneity in atypical WM during development. For this purpose, we utilized two independent cohorts, with 68 autistic children and 54 TDC in the discovery cohort, and 23 autistic children and 46 TDC in the replication cohort, ranging from 4 to 7 years of age. We constructed a developmental prediction modeling by using support vector regression (SVR) based on the typical development of WM microstructural properties of TDC to estimate the extent of WM atypical development in autistic children. Then, k-means clustering method was used to test whether subgroups that can represent different stages of atypical development existed. Finally, between-group differences in atypical WM microstructure and clinical manifestations were estimated in different subgroups of autistic children.

## Methods

### Participants

The discovery cohort included 77 autistic children and 63 TDC, and the replication cohort included 25 autistic children and 46 TDC. Autistic children were recruited from hospitals and rehabilitation institutions. TDC were recruited from local kindergartens. All the autistic children were diagnosed based on the fifth edition of the Diagnostic and Statistical Manual of Mental Disorders. Autistic trait was assessed by Autism Diagnostic Observation Schedule (ADOS) assessment. Autistic children were excluded if they (i) had psychiatric, neurological (e.g., epilepsy) or genetic (e.g., fragile X) disorders; (ii) had a history of loss of consciousness for more than 5 min; (iii) had a history of brain injury; and (iv) were under current medication with psychoactive drugs. TDC were recruited with the following criteria: (i) no neurological or psychiatric disorders; (ii) no history of severe medical problems; and (iii) not under psychotropic medications. After visual quality assurance, 3 TDC and 1 autistic child in the discovery cohort were excluded for low-quality DTI. Before applying matching algorithm, the handedness and sex were matched in the two groups. Then, we applied the greedy algorithm to create datasets that were not significantly different in age [42], yielding a discovery cohort of 68 autistic children and 54 age-, gender- and handedness-matched TDC, and a replication cohort of 23 ASD and 46 matched TDC (Table 1). Specifically, we deleted one participant at a time in the datasets that leave the rest data with the highest p value of the group difference of age, until the p value was higher than 0.1 (using Wilcoxon rank sum test).

**Table 1:** Demographics and clinical characteristics of the participants

	Discovery cohort						
	Autism			TDC			
	*N*	Mean ± SD	Range	*N*	Mean ± SD	Range	*p* value
Age (year)	68	5.46 ± 0.71	4.15–6.75	54	5.65 ± 0.67	4.25–6.70	0.13a
Sex	47 M/21F	–	–	33 M/21F	–	–	0.36b
Handedness	59R/3L/6 M	–	–	48R/1L/6 M	–	–	0.68b
Weight (kg)	68	19.90 ± 4.00	14–36	54	20.10 ± 3.50	14–31	0.51a
Height (cm)	68	111.95 ± 7.38	98–130	54	116.31 ± 7.57	95–131	0.0025a
Head motion (mm)	68	0.24 ± 0.05	0.16–0.40	54	0.35 ± 0.12	0.19–0.69	< 0.001a
Age of mother (year)	47	34.49 ± 5.84	25.70–53.06	36	35.28 ± 4.97	24.67–48.19	0.22a
ADOS total	60	18.40 ± 4.0	9–28	–	–	–	–

	Replication cohort						
	Autism			TDC			
	*N*	Mean ± SD	Range	*N*	Mean ± SD	Range	*p* value
Age (year)	23	5.13 ± 0.70	4.01–6.62	46	5.46 ± 0.80	4.04–6.88	0.13c
Sex	20 M/3F	–	–	39 M/7F	–	–	0.81b
Handedness	19R/4L	–	–	38R/8L	–	–	1 b
Head motion (mm)	23	0.10 ± 0.03	0.06–0.16	46	0.12 ± 0.09	0.06–0.54	0.30a
ADOS total	20	16.25 ± 5.4	7–26	–	–		–

aWilcoxon rank sum test (two-sided). b*χ*2 test. cTwo-sample t-test

*TDC*, typically developing children

The study of discovery cohort was approved by the research ethical committee of the University of Electronic Science and Technology of China (UESTC). The study of replication cohort was approved by the ethics review committee of Harbin Medical University (HMU). The guardians of each participant provided signed written informed consent after fully understanding the purpose of the study.

### Data acquisition

*Discovery cohort*. Whole-brain DTI was acquired at UESTC using a 3 T Discovery MR 750 scanner (GE Healthcare, Milwaukee, WI, USA) equipped with an eight-channel prototype quadrature birdcage head coil and echo planar imaging technique (repetition time = 8500 ms, echo time = 66.8 ms, field of view = 256 × 256 ${\mathrm{mm}}^{2}$, matrix size = 128 × 128, slice thickness = 2 mm, number of slices = 78, 60 diffusion-weighted volumes (*b* = 1000 s/${\mathrm{mm}}^{2}$) with four corresponding non-diffusion-weighted volumes (*b* value = 0 s/${\mathrm{mm}}^{2}$)). During the scan, autistic children were sedated by using 50 mg/kg chloral hydrate, which was administered by a trained and certified doctor, whereas TDC were instructed to watch cartoons while keeping their head still. For each participant, a caregiver was present during scanning.

*Replication cohort*. DTI was acquired at the Department of MR Diagnosis of the Second Hospital affiliated of Harbin Medical University using a 3 T Achieva scanner (Philips, Amsterdam, the Netherlands). The parameters of the spin echo (SE) based DTI sequence are: repetition time = 6100 ms, echo time = 93 ms, field of view = 256 × 256 ${\mathrm{mm}}^{2}$, matrix size = 128 × 128, slice thickness = 2 mm, number of slices = 78, 32 diffusion-weighted volumes (*b* = 1000 s/${\mathrm{mm}}^{2}$) with one corresponding non-diffusion-weighted volumes (*b* value = 0 s/${\mathrm{mm}}^{2}$). All of the participants were sedated using 50 mg/kg chloral hydrate which was administered by a trained and certified doctor during the scan, with a caregiver presented.

### Data processing

**Fig. 1:**
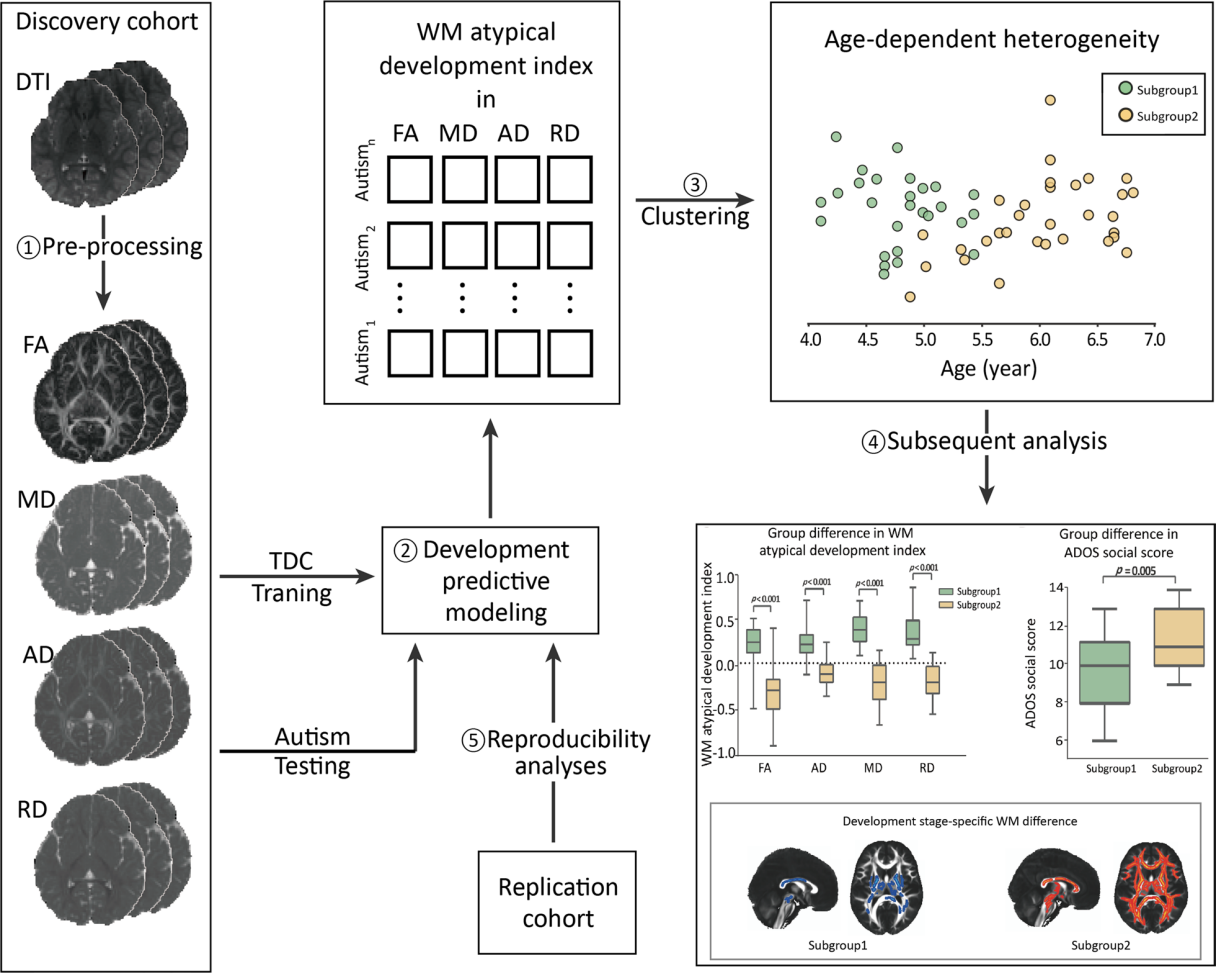
Data analysis flowchart. **1** DTI was pre-processed following the common FSL pipeline, creating FA, MD, AD and RD map of each participant. **2** Developmental prediction modeling was established by SVR using TDC dataset. For each autistic child, WM atypical development index was calculated. **3** The WM atypical development index matrix of autistic dataset was used as features in cluster analysis to investigate the age-dependent heterogeneity. **4** Subsequent analyses investigated the difference in WM atypical development index and autistic trait between developmental stages, and compared the difference of each developmental stage compared to TDC. *SVR*, support vector regression; *TDC*, typically developing children; *WM*, white matter

For each participant, images were pre-processed using FMRIB Software Library (FSL, https://fsl.fmrib.ox.ac.uk/fsl/fslwiki) by following the common pre-processing pipeline. First, a brain extraction tool [43] was applied to the first non-diffusion-weighted volume to generate a binary brain mask. Second, eddy current distortion and motion artifacts were corrected on the basis of the mask by using a FSL eddy tool [44]. Third, the diffusion tensor model was fitted at each voxel using ordinary linear least squares, by applying DTIFIT implemented within the FMRIB’s Diffusion Toolbox [45], creating each participant’s FA, MD, RD and axial diffusivity (AD) images (Fig. 1). Relative head motion was estimated using the FSL eddy quality control framework [46].

Voxel-wise statistical analysis was performed by using Tract-Based Spatial Statistics (TBSS), and all the analyses were performed in adult MNI-152 space [47]. First, the FA images of all participants were nonlinearly registered to the mean FA template of the IIT Human Brain Atlas (www.nitrc.org/projects/iit) [48] by employing FNIRT [49], which applies a b-spline representation of the registration warp field [50]. Second, a mean FA image was created and skeletonized. Third, each participant’s FA image was projected onto the FA skeleton image and merged into a 4D image for follow-up cross-subject statistics. Furthermore, each bundle’s mean FA value was extracted for each participant. These stages were repeated for MD, RD, and AD images. Here, bundles refer to the myelinated fiber tracts in WM [51].

### Exploration of the difference in WM diffusivity of autistic children compared to TDC using case–control analysis

To test whether any significant between-group difference in WM diffusivity existed, a case–control analysis was conducted by using two-sample t-test to explore the difference between the mean values of the four diffusivity characteristics between autistic children and TDC. Then, to test whether the relationship of age and WM diffusivity in autistic children was distinct from TDC, we calculated the Pearson correlation between the mean value of the diffusivity characteristic with age in TDC and autistic children, respectively.

### Developmental prediction modeling construction and WM atypical development index estimation

The SVR method is an ensemble learning method for regression which yields the output of an average prediction from multiple decision trees. First, the 54 TDC were randomly allotted into a training set and a testing set, with matched age, by using the scikit-learn package (Additional file 1: Supplemental Methods). Then, the SVR was utilized to train the development predictive model by using the training set, which predicted age using each diffusivity properties (i.e., FA, MD, RD, and AD) [52]. Furthermore, to adjust the age-related bias introduced by the model [53], the relationship between the true value and the difference value between the predicted and true values of each subject in the testing set was calculated as follows:

$${\text{deviation}} = - \left( {\alpha \times \Omega + \beta } \right),$$

where $\Omega$ is the true age, the deviation represents the difference between the predicted and true values, and $\alpha$ and $\beta$ stand, respectively, for the slope and intercept of the linear regression model. Finally, the model was tested on 68 autistic children. Each participant’s age-related deviation in the four diffusivity properties was calculated separately. Then, the difference between the age-related deviation and the bias of each subject was calculated to acquire the bias-free age-related deviation, which is regarded as the WM atypical development index (Fig. 1). This index can be described as follows:

$${\text{deviation}}_{{{\text{bias}}\;{\text{free}}}} = {\text{deviation}} - \left( {\alpha \times \Omega + \beta } \right).$$

Model performance was evaluated by using the fivefold cross-validation procedure. At each time, we used 4 of the 5 folds to train the model, and the mean squared error (MSE) and the mean absolute percent error (MAPE) were estimated on the remaining portion of the data. This process would be repeated 5 times until all data have been tested once. MSE measures the average of the squares of the absolute error between the predicted and true values, whereas MAPE is the average of relative error in terms of percentages.

### Age-dependent heterogeneity of autistic children

The matrix of the WM atypical development index (68 subjects, 4 features) measured on the basis of four diffusivity properties was used to explore age-dependent heterogeneity across autistic children by applying k-means clustering analysis. The silhouette coefficient was calculated to determine the optimal number of clusters, which varied from 2 to 10. According to the silhouette criteria, the optimal number of clusters was 2 [54]. Therefore, two subgroups of autistic children were found (Fig. 1).

We firstly applied two-sample t-test to estimate the difference between the two subgroups of autistic children on the basis of demographic information (i.e., age, sex, and handedness), WM atypical development index, and ADOS scores. Eight subjects were excluded from the autistic trait estimation due to the loss of ADOS scores. Then, the relationship of the WM atypical development index with age was estimated by using partial correlation, and the significance level was set to *p* < 0.05 after false discovery rate (FDR) correction.

### Developmental stage-specific difference of WM microstructure in autistic children

The randomize tool [55] implemented in FSL was used for the group comparison of each subgroup of autistic children with the rematched subsets of TDC to delineate the developmental stage-specific difference in WM microstructures. Analysis was performed by using the threshold-free cluster enhancement-based two-sample t-test with 5000 permutations in the presence of nuisance variables (i.e., age, gender, handedness and relative head motion), wherein the significance level was set to *p* < 0.05 (family-wise error corrected).

### Reproducibility analysis

Because there was a between-group difference in head motion in the discovery cohort, we conducted reproducibility analyses to verify the robustness of our findings: (1) using features that regressed out head motion; (2) using the replication cohort, in which there were no group differences in head motion. First, the developmental prediction modeling was constructed using the features controlling for nuisance variance, including gender, handedness and particularly the head motion. Subgroups of the autistic children were identified using k-means clustering method. Age, WM atypical index and ADOS scores were compared between the two subgroups using two-sample t-test, and the correlation of the WM atypical development index with age was calculated using Pearson correlation. Second, to exclude the effect of head motion on the results of age-dependent heterogeneity in autistic children, we conducted a reproducibility analysis using a replicated cohort with no between-group differences in head motion. Besides, we utilized random forest regression (RFR) rather than SVR to construct the developmental prediction modeling, to validate the finding that the age-dependent heterogeneity of autistic children is not model-dependent.

## Results

### WM atypical development index revealed age-dependent heterogeneity in autistic children

**Fig. 2:**
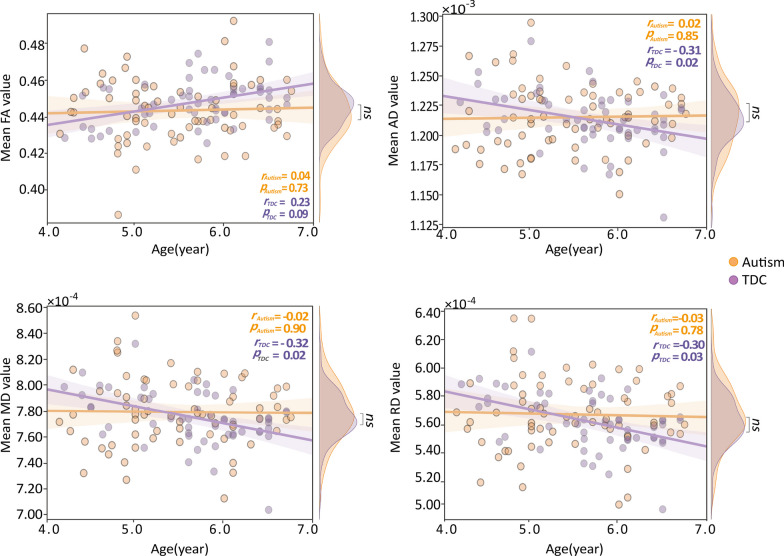
Relationship of age and WM diffusivity in autistic children and TDC. Mean values of the four diffusivity characteristic were not significantly different between autistic children and TDC. Orange lines represent the linear fitting straight line of autistic children, while the purple lines represent the TDC. Orange nodes represent autistic children, while purple nodes represent the TDC. *TDC*, typically developing children; *NS*, not significant

We first applied the two-sample t-test to test whether any between-group difference existed between autistic children and TDC in terms of the mean values of the four WM diffusivity characteristics. No significant between-group difference was found on the four WM diffusivity characteristics. Correlation analysis revealed significant correlations between age and the mean AD, RD, and MD values (${r}_{\mathrm{AD}}$ = − 0.31, *p* = 0.02; ${r}_{\mathrm{RD}}$ =  − 0.30, *p* = 0.03; ${r}_{\mathrm{MD}}$ = − 0.32, *p* = 0.02) and a marginally significant correlation between age and mean FA value (${r}_{\mathrm{FA}}$ = 0.23, *p* = 0.09) in TDC. Furthermore, we found that autistic children presented a flat development trajectory of WM diffusivity. However, we did not find any significant associations between WM diffusivity and age in autistic children (Fig. 2).

**Fig. 3:**
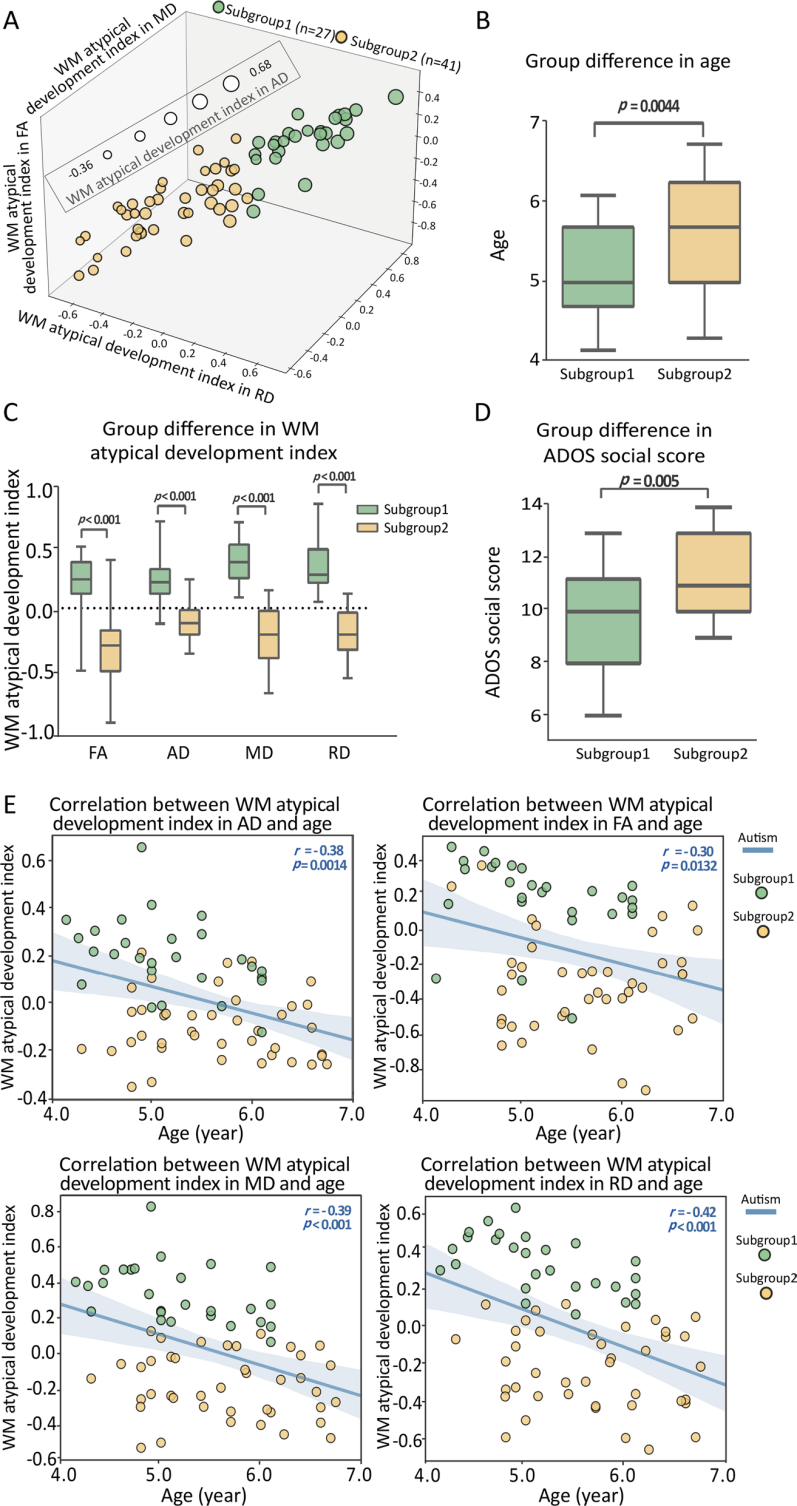
Age-dependent heterogeneity and its relationship and distinct distribution in clinical autistic trait. **A** Visualization of the two subgroups. **B** Group difference in age. **C** Group difference in age WM atypical development index. **D** Group difference in ADOS social score. **E** Age was associated with WM atypical development index. Blue lines represent the linear fitting of autistic children, while the green nodes represent subgroup 1 of autistic children, and yellow nodes belongings to subgroup 2 of autistic children. *TDC*, typically developing children; *WM*, white matter

The SVR model performances (i.e., MSE and MAPE) of the four diffusion properties FA, MD, RD, and AD were 0.68 and 11.89%, 0.61 and 11.29%, 0.58 and 11.01%, and 0.69 and 11.99%, respectively. By using the k-means clustering method, we clustered the subjects into two subgroups (Fig. 3A) exhibiting significant distinct development stages (${t}_{\mathrm{Age}}$ = − 2.95, *p* = 0.0044) (Fig. 3B). We did not find significant differences in sex and handedness between the two stages. Moreover, we found significant differences in the WM atypical development index (${t}_{FA}$ = 7.50, *p* < 0.001; ${t}_{\mathrm{AD}}$ = 8.20, *p* < 0.001; ${t}_{\mathrm{RD}}$ = 11.71, *p* < 0.001; ${t}_{\mathrm{MD}}$ = 11.80, *p* < 0.001; FDR corrected, number of tests = 4) between the two stages (Fig. 3C). We also found significant difference in ADOS social score (*t* =  − 2.92, *p* = 0.005) between the two stages, where the autistic children in subgroup 2 showed slightly higher level of autistic trait than those in subgroup 1 (Fig. 3D). In addition, we found that age was negatively associated with the WM atypical development index in FA (*r* =  − 0.30, *p* = 0.0132), AD (*r* =  − 0.38, *p* = 0.0014), MD (*r* =  − 0.39, *p* < 0.001), and RD (*r* =  − 0.42, *p* < 0.001) (FDR corrected, number of tests = 4). Besides, we found a period of high developmental diversity between the ages of 5 and 6 years, because almost all of the autistic children younger than 5 years of age were in subgroup 1, and almost all of those older than 6 years of age were in subgroup 2 (Fig. 3E).

### Developmental stage-specific difference of WM microstructure in autistic children

**Fig. 4:**
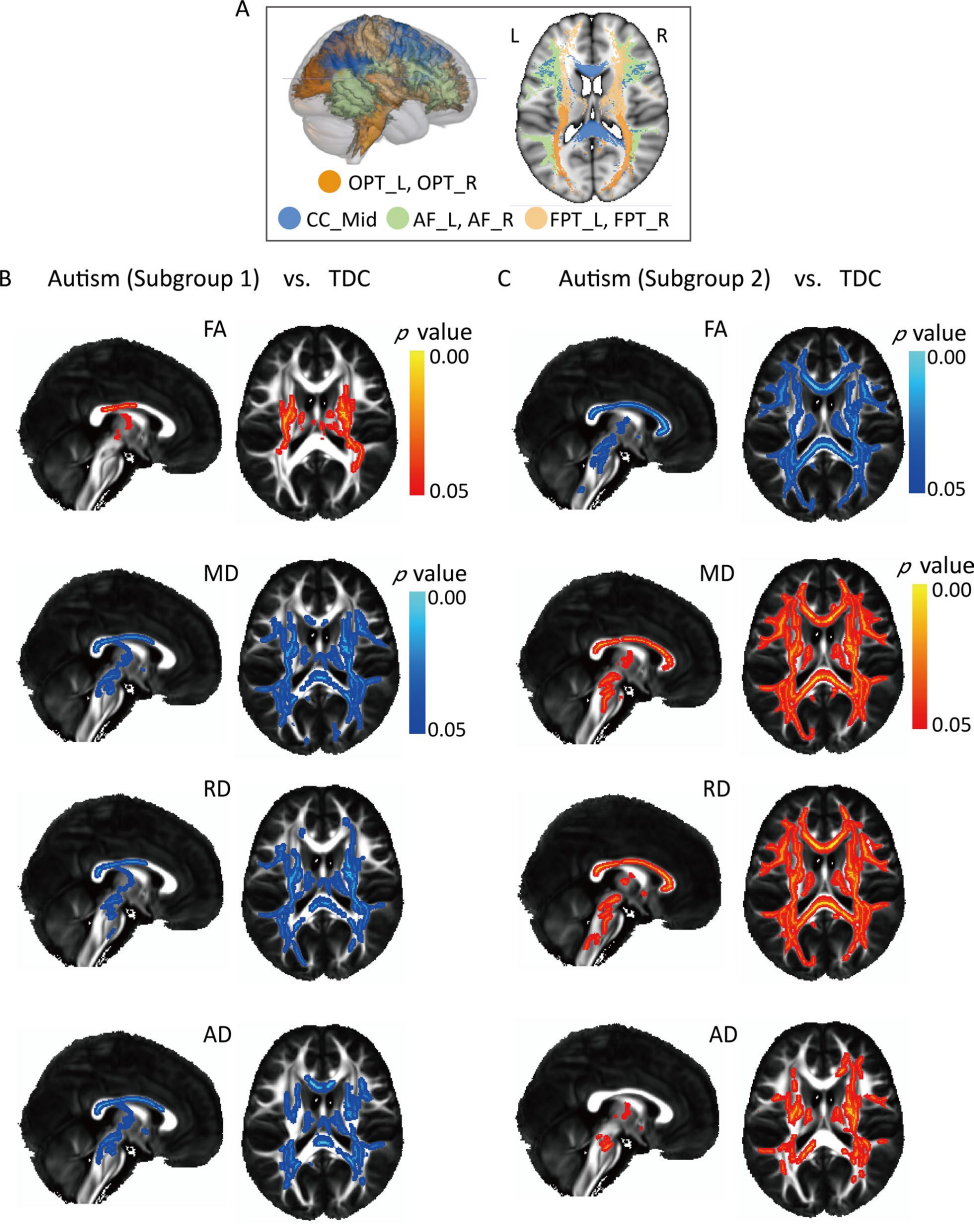
Developmental stage-specific difference in WM microstructure. **A** Visualization of significantly different bundles between autistic children and TDC. **B** Significantly increased FA and decreased MD, RD and AD in CC_Mid, bilateral AF, OPT and FPT were found in subgroup 1 compared to TDC (*p* < 0.05, family-wise error corrected). **C** Significantly decreased FA and increased MD, RD and AD in those bundles were found in subgroup 2 compared to TDC (*p* < 0.05, family-wise error corrected). *WM*, white matter; *CC*_Mid, middle of corpus callosum; *AF*, arcuate fasciculus; *OPT*, occipitopontine; *FPT*, frontopontine; *TDC*, typically developing children

By using TBSS and after controlling for age, gender, handedness, and relative head motion, we found autistic children in the two subgroups showed a reverse pattern of atypical WM diffusivity. Subgroup 1 displayed significantly increasing FA and decreasing MD, RD and AD in the bilateral arcuate fasciculus (AF), bilateral frontopontine (FPT), bilateral occipitopontine (OPT), and middle of the corpus callosum (CC), whereas subgroup 2 presented significantly increasing MD, RD, and AD and decreasing FA in these bundles (*p* < 0.05, family-wise error corrected) (Fig. 4). We also extracted the value of significantly atypical bundles and drew the relationship of age and WM diffusivity in autistic children and TDC, all of which exhibited an atypical relationship with age in autistic children (Additional file 1: Figure S1).

### Reproducibility of findings

The developmental prediction modeling was constructed by using features controlling for head motion, yielding similar results of significant group differences in age and WM atypical development index, and association between age and WM atypical development index. According to the silhouette criteria, the optimal number of clusters in k-means clustering analysis was also 2. We still clustered subjects into two subgroups, which showed significantly distinct development stages (${t}_{\mathrm{Age}}$ = − 3.62, *p* < 0.001) (Additional file 1: Figures S2A and S2B). We also found a significant difference in the WM atypical development index (${t}_{\mathrm{FA}}$ = 5.03, *p* = 0.017; ${t}_{\mathrm{AD}}$ = 4.74, *p* < 0.001; ${t}_{\mathrm{RD}}$ = 10.46, *p* < 0.001; ${t}_{\mathrm{MD}}$ = 12.22, *p* < 0.001; FDR corrected, number of tests = 4) between two subgroups (Additional file 1: Figure S2C). In addition, we found a significant difference in ADOS social score (*p* = 0.05) between the two subgroups (Additional file 1: Figure S2D). Furthermore, we found that age was negatively associated with the WM atypical development index in FA (*r* =  − 0.27, *p* = 0.036), AD (*r* =  − 0.26, *p* = 0.036), MD (*r* =  − 0.44, *p* < 0.001), and RD (*r* =  − 0.50, *p* < 0.001) (Additional file 1: Figure S2E, FDR corrected, number of tests = 4).

Second, the development predictive modeling was reproduced using the replication cohort, in which there were no group differences in head motion, and similar results of age-dependent heterogeneity in autistic children were found. In the k-means clustering analysis, the optimal number of clusters also resulted to be 2, so we still clustered subjects into two subgroups, which showed significantly distinct development stages (${t}_{\mathrm{Age}}$ = − 2.40, *p* = 0.0256) (Additional file 1: Figures S3A and S3B). We still found a significant difference in the WM atypical development index (${t}_{\mathrm{FA}}$ = 1.44, *p* = 0.07; ${t}_{\mathrm{AD}}$ = 7.37, *p* < 0.001; ${t}_{\mathrm{RD}}$ = 6.99, *p* < 0.001; ${t}_{\mathrm{MD}}$ = 6.25, *p* < 0.001; FDR corrected, number of tests = 4) between two subgroups (Additional file 1: Figure S3C).

Moreover, we applied RFR to rebuild the model to validate that the findings are not model-dependent. Considering that the optimal number of clusters was also 2, we divided the subjects into two developmental subgroups (${t}_{\mathrm{Age}}$ = − 3.15, *p* = 0.0025) (Additional file 1: Figures S4A and S4B). We still found a significant difference in the WM atypical development index (${t}_{\mathrm{FA}}$ = 8.94, *p* < 0.001; ${t}_{\mathrm{AD}}$ = 6.57, *p* < 0.001; ${t}_{\mathrm{RD}}$ = 13.78, *p* < 0.001; ${t}_{\mathrm{MD}}$ = 10.76, *p* < 0.001; FDR corrected, number of tests = 4) between two subgroups (Additional file 1: Figure S4C). In addition, we found a significant difference in ADOS social score (*t* =  − 2.28, *p* = 0.026) between the two subgroups (Additional file 1: Figure S4D). Moreover, we discovered that age was negatively associated with the WM atypical development index in FA (*r* =  − 0.34, *p* = 0.005), AD (*r* =  − 0.40, *p* < 0.001), MD (*r* =  − 0.43, *p* < 0.001), and RD (*r* =  − 0.52, *p* < 0.001) (Additional file 1: Figure S4E). In general, these findings, which were estimated in different conditions, were robust and constantly showed the developmental deviation in autistic children while comparing with TDC.

## Discussion

In this study, we constructed a developmental prediction modeling to estimate the WM atypical development index of autistic children and clustered autistic children into two subgroups representing different developmental stages. We found differences in clinical manifestations between subgroups and revealed developmental stage-specific difference in autistic children. Our results enhanced our understanding of the age-dependent heterogeneity of autistic children from a neuroimaging perspective and delineated the atypical pattern of WM microstructures in different developmental stages of autistic children. Moreover, the current study pointed out the importance of disentangling the age-dependent heterogeneity in autistic children across early childhood.

### Age-dependent heterogeneity in autistic children and distinct clinical manifestation

Previous studies have suggested that WM volume and diffusion properties exhibited a highly relevant pattern of developmental change, showing a rapid increase in FA and decrease in MD, RD, and AD in early childhood [56, 57]. In line with the relationship of age and WM diffusivity we depicted in the current study, autistic children have been found to present an early overgrowth pattern followed by slower development compared with TDC [22, 23, 58]. In addition, the non-significant difference in WM diffusivity between autistic children and TDC further verified our hypothesis that the atypical WM diffusivity in autistic children might be clouded by age-dependent heterogeneity within groups, which cannot be revealed by simply conducting a case–control analysis.

Furthermore, we found a significantly negative association between age and the WM atypical development index, with the lager positive index referred to more accelerated growth, and the lager negative one referred to more delayed growth. We divided autistic children into two subgroups in terms of WM atypical development index, which showed distinct developmental stages. We found different clinical manifestation between the two subgroups, wherein subgroup 2 exhibited higher ADOS social score. A previous study on autistic children reported that behavioral intervention, per unit time, is more efficient for younger children (aged 2–5.15 years) than for older children (aged 5.15–7.14 years) [59]. The higher level of autistic trait observed in subgroup 2 might be attributable to the weakening effect of interventions. In line with a review of evidence base for “earlier is better” with regard to interventions for young children with ASD [60], a study reported that the positive effects of treatment diminished after age of 3.81 years in autism [61]. This observation needs further validation using longitudinal data. Our results further proved the assumption that during childhood, autistic children exhibit accelerated growth followed by a period of delay [62, 63] and provided a complete picture of WM microstructure development.

Moreover, we estimated the individual deviation in autistic children and clustered them into two subgroups that revealed a period of high developmental diversity between the ages of 5 and 6 years. Myelination in typical brain undergoes a rapidly advance till the age of 5 and continues with a slower process till the third decade of life [64]. A neuroimaging study provided in vivo evidence showing age-related effects between TDC and autistic children at approximately 5 years of age and found a significant age-related increase in myelin content in TDC but not in autistic children [65]. Likewise, another study found a significant age-related interaction in WM volume in TDC, but not in autistic children, delineating an age-related increase in WM volume and age-related effects at approximately 6 years of age [62]. Overall, in autistic children, the age range of 5–6 years is a developmental and varied period before which premature brain development is reported and after which brain development presents an atypically slowed pattern. Our results enhanced our understanding of the protracted growth of WM in autistic children, and provided a neuroimaging footing from which to gain insights into the age-dependent heterogeneity of autistic children.

Because of the between-group difference in head motion in the discovery cohort, we conducted reproducibility analyses to verify the results we found were independent from head motion. Compared to the original findings, the reproducibility analyses yielded similar results of significant group differences in age and WM atypical development index, both of which delineated age-dependent heterogeneity in autistic children. This might be explained by, after establishing the developmental prediction modeling, the analyses of age-dependent heterogeneity in autistic children were conducted within the autistic group, and we did not find any differences in head motion between subgroup 1 and subgroup 2 in the original findings (*t* =  − 0.84, *p* = 0.40). Besides, we performed the group comparison of each subgroup of autistic children with the rematched subsets of TDC while controlling for head motion. Overall, the reproducibility analyses further validated our findings of age-dependent heterogeneity in autistic children.

### Difference in WM microstructure between two subgroups

We delineated the developmental stage-specific difference of WM microstructures in autistic children, revealing an atypical overgrowth pattern in subgroup 1 and delayed development in subgroup 2, with subgroup 1 comprising autistic children aged under 6.5 years and subgroup 2 comprising those aged more than 5.5 years. Previous studies have reported significantly increased FA and decreased AD, MD, and RD in young autistic children (< 5 years old) [66–69] and reverse patterns in older autistic children (> 6 years old) [70–73]. Here, our findings further delineated the flatter development of WM diffusivity in autistic children than in TDC, leading to this reverse phenomenon. The precocious maturation of WM might reflect fetal axon excess in autistic children [74]. It might suggest the weakened regulation of neurogenesis in younger autistic brain, in line with a recent study of autism genes reporting vulnerability in neurogenesis [75]. Whereas, the delayed maturation might be driven by delayed myelination [65]. It is accordant to a previous study, in which combined fetal inflammation and postnatal hypoxia resulted in delayed cortical myelination and autism-like behavior in rat model [76]. Our findings bore upon the inconsistent reports surrounding WM microstructures in autistic children and stressed the importance of the developmental period as a crucial factor for the heterogeneity of WM maturation.

Our study validated the reverse patterns observed across two developmental stages by clustering the WM atypical development index in autistic children. We found significant difference in AF in both developmental stages. AF is critically involved in human language processing [77, 78]. Atypical diffusion property in AF and language challenges have been frequently reported in autistic people [79–84]. However, neuroimaging studies on atypical diffusion property in AF in autistic people have reported inconsistent results, including left-only, right-only, and bilateral difference in AF [18]. Our findings provided a developmental perspective of the atypical diffusion property in AF in autistic people. CC was another atypical fiber we found throughout the two stages. Atypical CC in autistic people, including atypical morphometry and diffusion properties, has been studied for decades [85–89]. CC is the main fiber conducting interhemispheric signals [90]. The atypical diffusion property in CC would cause disrupted connectivity between the two hemispheres. Atypical interhemispheric connectivity and its association with social challenges in autistic people have been reported in former studies [91, 92]. Our findings added evidence of the atypical diffusion property in CC and delineated the change of MD, RD and AD in CC from atypically reduced to increase during development.

Furthermore, we found inversely atypical diffusion properties between the two stages in bilateral FPT and bilateral OPT, both of which are parts of the corticopontine fibers. The corticopontine fibers are crucial pathways mediating information transmission from the cerebral cortex to the cerebellum, and conducting impulses from the cerebral cortex to pontine nuclei then to the cerebellum through pontocerebellar projection [93, 94]. Neuroimaging studies have implicated atypical cortico-cerebellar system in autistic people [95, 96]. Specifically, the disruption of the fronto-cerebellar circuit might induce the disturbance of eye movement and motor operation [97, 98], which are characteristics in autistic people, especially in younger autistic children [99–101].

### Methodological considerations

In the present study, we constructed the developmental prediction modeling using four widely used diffusion tensor-based parameters. Among these parameters, FA measures the degree of diffusion anisotropy [102]; MD measures the overall magnitude of diffusion irrespective of the direction [103]; and AD and RD represent the water diffusivities parallel and perpendicular to axonal fibers, respectively [104]. These four parameters represent diverse information while reserving the consistency of diffusivity, making evaluating the WM microstructure from different aspects feasible. Moreover, WM microstructures have been reported to be widely atypical in autistic children [18, 105] and have demonstrated a significant relationship with age in TDC [106]. Thus, these parameters could capture the age-dependent information of typical development in TDC and the neurodiversity-sensitive information of atypical WM microstructure in autistic children at the same time. In the present study, by combining these diffusion properties, we were able to reveal the age-dependent heterogeneity of autistic children through a data-driven way.

In addition, we selected SVR because it is suitable for small sample size problem [107–109]. Furthermore, the bias introduced by predictive modeling has been found to be age-dependent, and the predicted value is overestimated in younger subjects and underestimated in older ones [110]. We selected the age-adjusted method proposed by Beheshti et al. because it reduces the variance of the deviation and results in a low mean absolute error after bias adjustment [111].

## Limitation

In the present study, we delineated the age-dependent heterogeneity by using developmental prediction modeling. However, several limitations should be noted. First, due to the relatively small amounts of autistic females, we did not separate the participants into female and male groups to construct the developmental prediction modeling. Future studies with a large sample size should take the gender difference into consideration. Second, the observation in this study focused only on the pre-school autistic children. Further studies on the biological mechanism of autistic people from later childhood to early adolescence are necessary. Third, given that we used the datasets limited to the Chinese population, the generalizability of our results to other ethnic populations remains unclear. Thus, future work on different ethnic populations is needed to validate our findings. Fourth, sampling bias might present because an association between age and ADOS score was found in the discovery cohort. Future study with a large sample size is needed to confirm the results of the present study. Fifth, given that the ethnicity, socioeconomic status of family and siblings might influence the early development of children, future study is needed to collect those data and investigate their association with atypical WM in autism. Furthermore, given that we used the cross-sectional datasets, future study of longitudinal data is needed to confirm the age-dependent heterogeneity of autistic children.

## Conclusion

In the present study, we proposed neurobiologically interpretable indexes by constructing a developmental prediction modeling and confirmed the contrasting nature of two developmental stages by using a data-driven method. Furthermore, our findings demonstrated that individual deviation of pre-school autistic children reduced with age and contributed to the neurodiversity of autistic people. We elucidated the progressive change in WM in autistic children and drew attention to the elucidation of the age-dependent heterogeneity of autistic children before follow-up analyses.

### Supplementary Information

**Additional file 1:** Supplementary Methods.

## Data Availability

The datasets generated and/or analyzed during the current study are not publicly available due to privacy concerns for minors.

## Abbreviations

TDC: Typically developing children
DTI: Diffusion tensor imaging
ADOS: Autism Diagnostic Observation Schedule
WM: White matter
FSL: FMRIB Software Library
FA: Fractional anisotropy
MD: Mean diffusivity
RD: Radial diffusivity
AD: Axial diffusivity
SVR: Support vector regression
MSE: Mean squared error
MAPE: Mean absolute percentage error
