# Supplementary material for: Developmental prediction modeling based on diffusion tensor imaging uncovering age-dependent heterogeneity in early childhood autistic brain
Source: Mol Autism. 2023 Oct 30;14:41. doi: 10.1186/s13229-023-00573-2 (PMC10614412; doi:10.1186/s13229-023-00573-2)
Supplement: Supplementary file 1 — Additional file 1: Supplementary Methods. [file 13229_2023_573_MOESM1_ESM.docx]

**Supplementary Materials**

**Supplemental Methods**

We used stratified k-folds algorithm implemented in Scikit-learn package (https://scikit-learn.org) to divide the data into training and test sets, while at the same time matching the age between the two sets. First, we sorted the subjects according to the age. Then, each subject was assigned to a label from 0 to 3 (i.e. from younger to older), which means subjects were separated into four classes according to their age. Finally, based on the labels, the stratified k-folds algorithm was applied to split the data into two clusters that containing the same distribution of classes. We initially used cluster 1 as the training set, with cluster 2 used as the testing set.


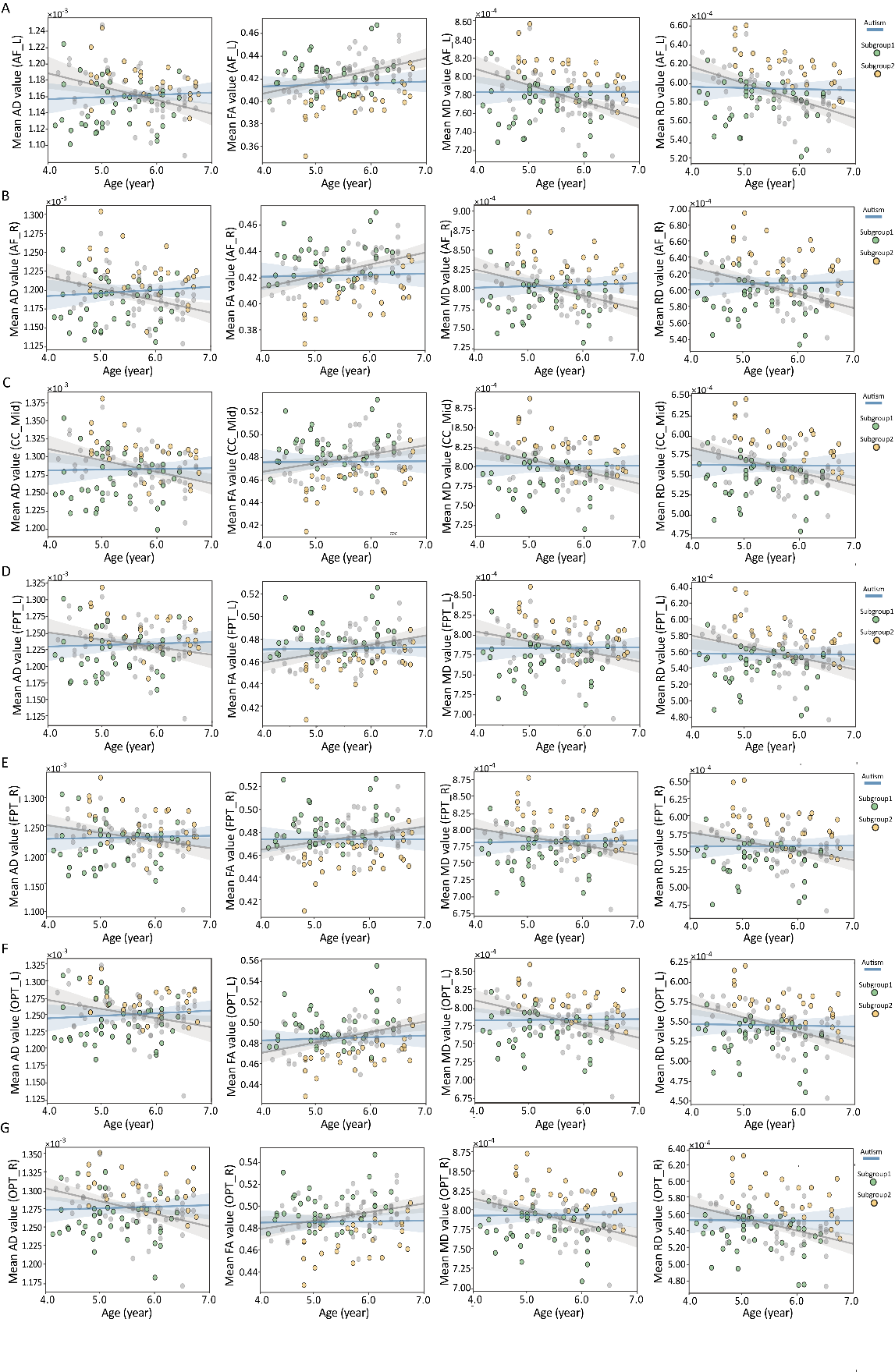
**Supplemental Figures**

**Figure S1. Relationship of age and WM diffusivity of significantly atypical bundles.** Blue lines represent the linear fitting straight line of autistic children, while the gray lines represent the TDC. Green nodes represent subgroup 1 of autistic children, and yellow nodes belongings to subgroup 2 of autistic children, while gray nodes represent the TDC. CC_Mid, middle of corpus callosum; AF, arcuate fasciculus; OPT, occipitopontine; FPT, frontopontine; TDC, typically developing children.


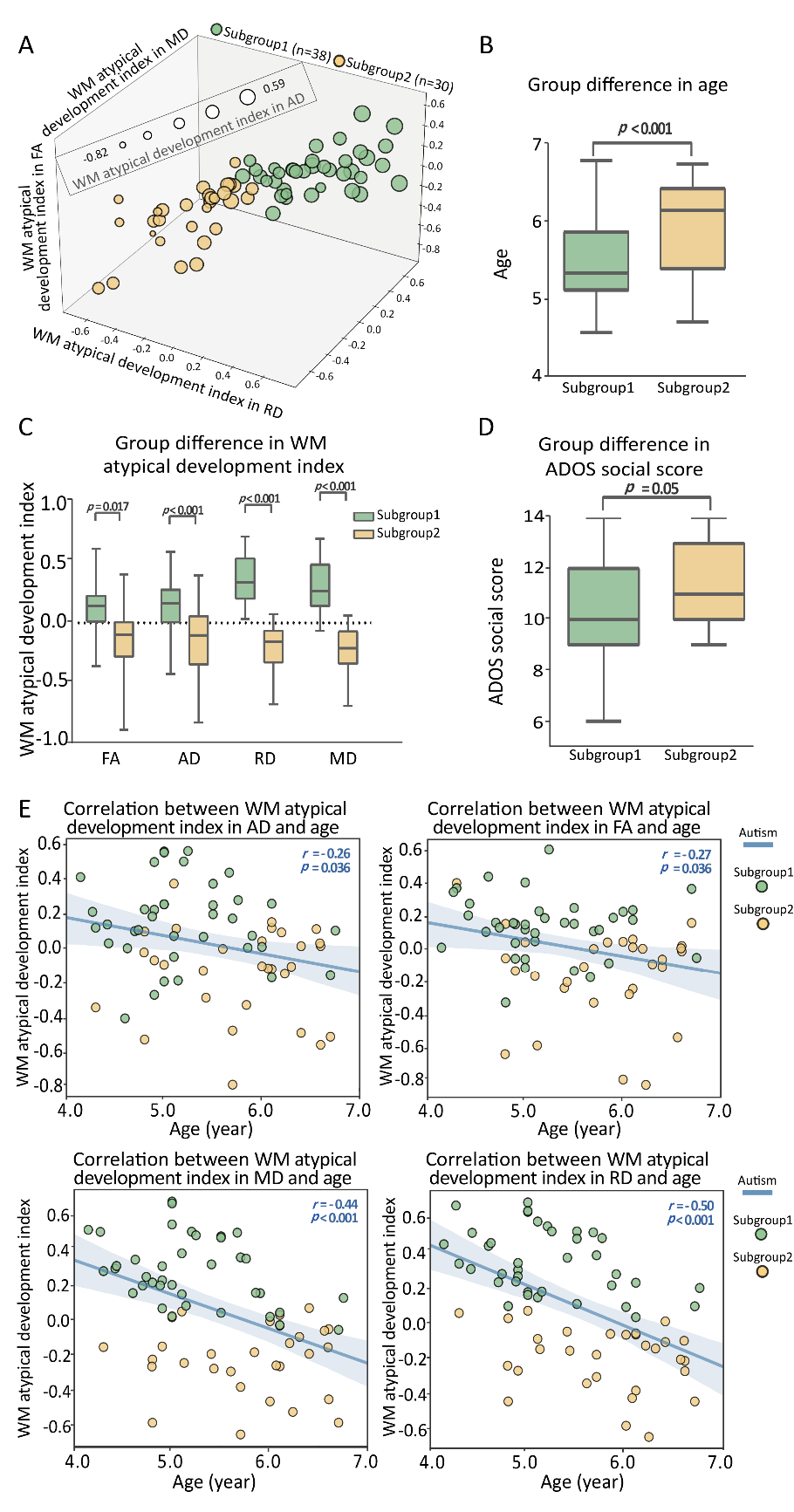


**Figure S2. Reproducibility of findings using features controlling for nuisance variance. (A) Visualization of the two subgroups. (B) Group difference in age. (C) Group difference in age WM atypical development index. (D) Group difference in ADOS social score. (E) Age was associated with WM atypical development index.** Blue lines represent the linear fitting of autistic children, while the green nodes represent subgroup 1 of autistic children, and yellow nodes belongings to subgroup 2 of autistic children. TDC, typically developing children; WM, white matter.


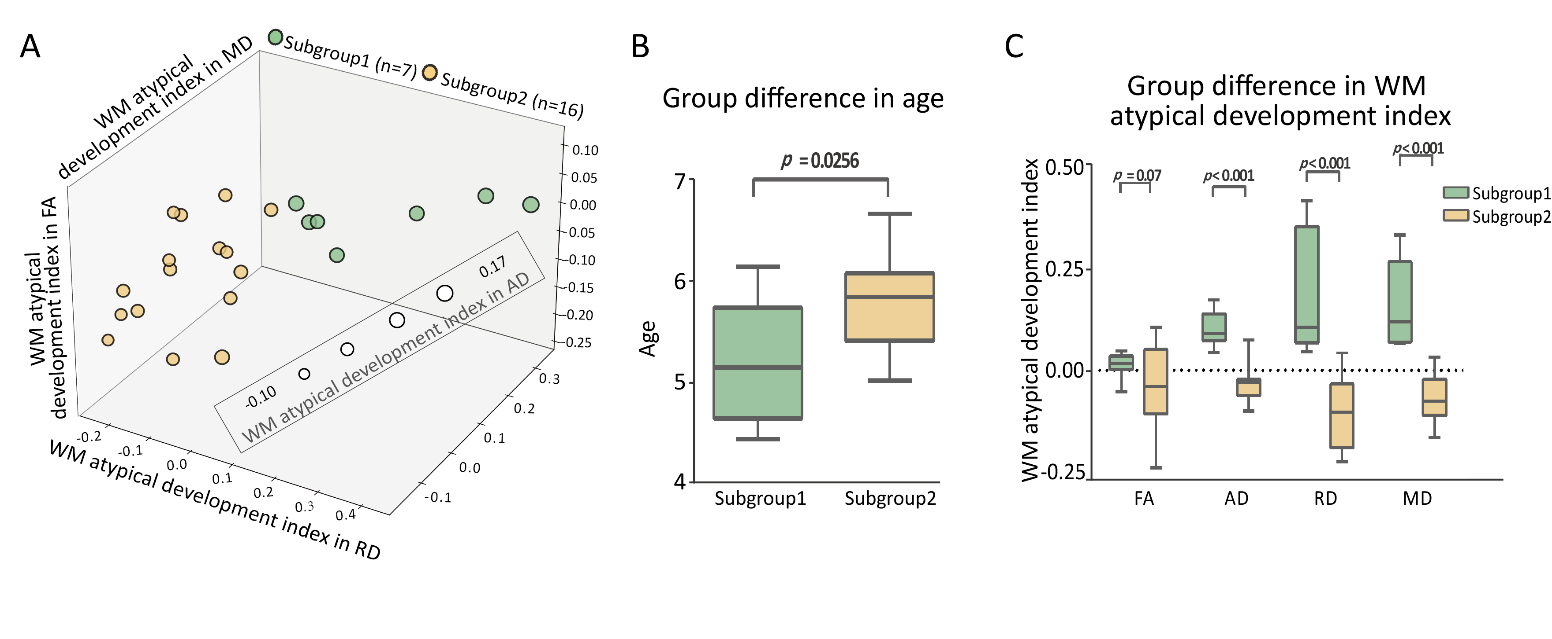


**Figure S3. Reproducibility of findings using replicated cohort. (A) Visualization of the two subgroups. (B) Group difference in age. (C) Group difference in age WM atypical development index.** Blue lines represent the linear fitting of autistic children, while the green nodes represent subgroup 1 of autistic children, and yellow nodes belongings to subgroup 2 of autistic children. TDC, typically developing children; WM, white matter.


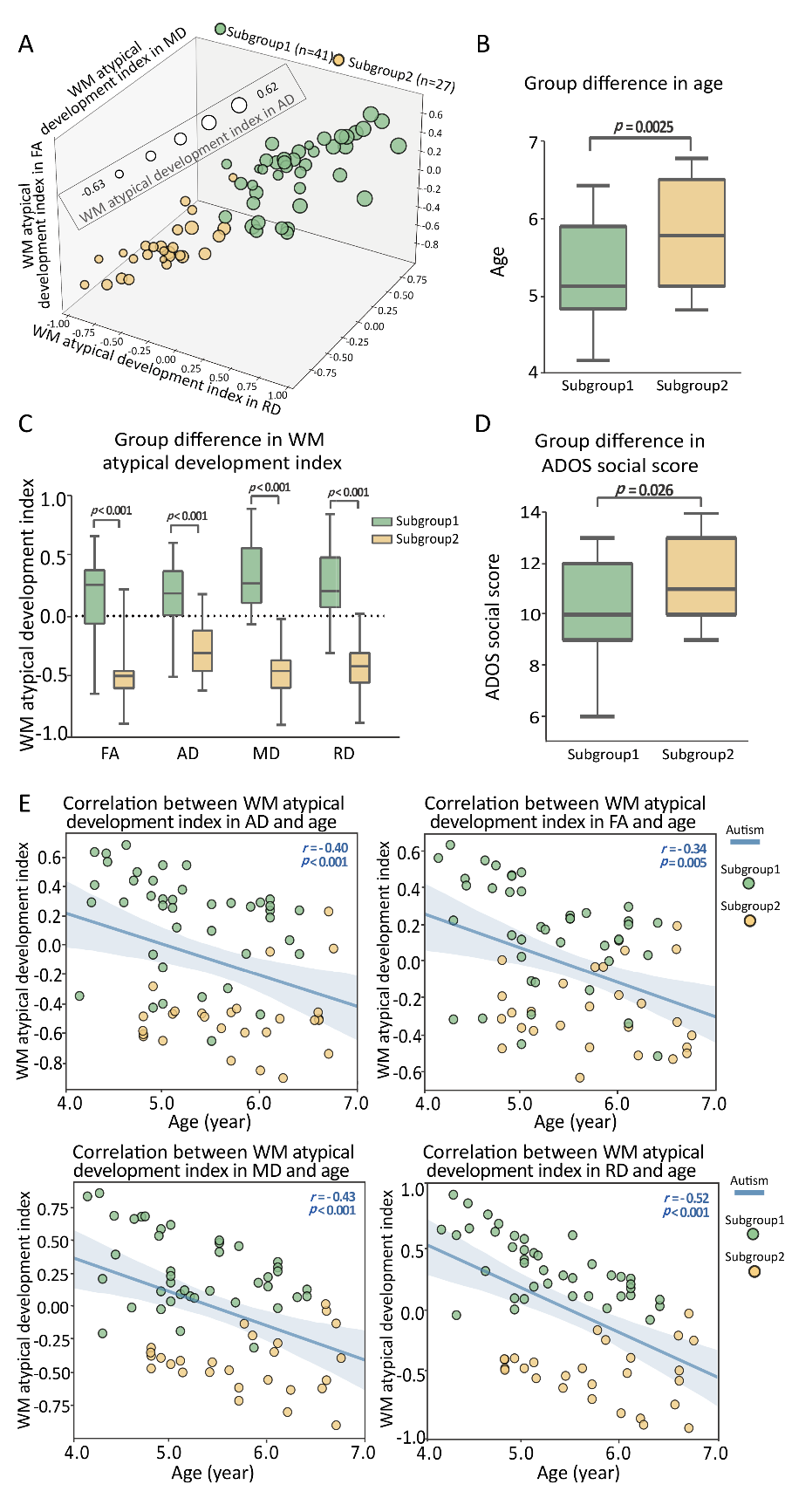


**Figure S4. Reproducibility of findings using RFR. (A) Visualization of the two subgroups. (B) Group difference in age. (C) Group difference in age WM atypical development index. (D) Group difference in ADOS social score. (E) Age was associated with WM atypical development index.** Blue lines represent the linear fitting of autistic children, while the green nodes represent subgroup 1 of autistic children, and yellow nodes belongings to subgroup 2 of autistic children. TDC, typically developing children; WM, white matter; RFR, random forest regression.
